# Efficacy and safety of atomoxetine in the treatment of ADHD in children and adolescents: a systematic review

**DOI:** 10.3389/frcha.2025.1731330

**Published:** 2026-01-06

**Authors:** Edmundo Alberto Barbosa Bastos De Souza Neto, Halley Ferraro Oliveira, Williams Santos Ramos, Estelio Henrique Martin Dantas, Barbara Hellen Alves Pereira, Rebeca de Souza Mariano Bastos

**Affiliations:** 1 Department of Medicine, Universidade Tiradentes, Aracaju, Brazil; 2 Independent Researcher, São Paulo, Brazil; 3 Department of Medicine, Universidade Brasil, São Paulo, Brazil; 4 Medical Residency Program, Hospital de Urgências de Sergipe Governador Joäo Alves Filho, Aracaju, Brazil

**Keywords:** ADHD, adolescents, atomoxetine, safety, efficacy, pharmacological treatment, adverse effects

## Abstract

**Objective:**

To evaluate the efficacy and safety of atomoxetine in the treatment of adolescents with ADHD, comparing its effects with other available treatments. The primary outcome was the reduction of ADHD symptoms, and the secondary outcome was the occurrence of adverse effects.

**Methods:**

A search was conducted in the Medline/PubMed, EMBASE, and Web of Science databases. The research question and strategy were based on the PICO model. Inclusion criteria were restricted to studies involving children and adolescents aged 6–16 years, focusing on comparisons between atomoxetine and other treatments. Eligible studies were published in English, Spanish, or Portuguese, with no restrictions on publication year. A total of 575 articles were initially retrieved. After removing duplicates, 527 references were screened by title and abstract, and 69 were selected for full-text review. Following this stage, 63 references were excluded, and 6 studies were ultimately deemed eligible.

**Results:**

The six included studies involved a total of 905 participants. Atomoxetine demonstrated comparable efficacy to methylphenidate in reducing ADHD symptoms. The most common adverse effects were nausea, fatigue, and appetite changes. No severe adverse events were consistently reported. Atomoxetine's efficacy was particularly evident in patients who did not tolerate or respond to stimulant medications.

**Conclusion:**

Available evidence suggests that atomoxetine is an effective and safe option for treating ADHD in adolescents, representing a valid alternative particularly for patients who do not tolerate stimulant medications. Continued research, especially long-term studies, is necessary to confirm its efficacy across different patient subgroups.

**Systematic Review Registration:**

Identifier CRD420251152121.

## 1 Introduction

Attention-Deficit/Hyperactivity Disorder (ADHD) is a prevalent neuropsychiatric disorder characterized by persistent symptoms of inattention, hyperactivity, and impulsivity that impair academic, social, and emotional functioning. Studies conducted in Latin America have reported wide variability in the prevalence of ADHD across different populations, as described in the literature (1). In the United States, the prevalence is approximately 12% among children and adolescents (2).

The diagnosis of ADHD is based on clinical criteria established by the *Diagnostic and Statistical Manual of Mental Disorders, Fifth Edition* (DSM-5) (3), which extended the age limit for symptom onset from 7 to 12 years, resulting in an adjusted prevalence rate of 10.84% (4). ADHD is recognized for its clinical heterogeneity and frequent comorbidity with other psychiatric disorders such as anxiety, depression, and conduct disorders, making its therapeutic management particularly challenging.

ADHD treatment is multimodal, involving both pharmacological and psychosocial interventions. Stimulant medications, such as methylphenidate and amphetamines, are considered first-line treatments due to their proven efficacy in reducing the core symptoms of the disorder (5). Methylphenidate, in particular, is widely used in children and adolescents and has been approved for use in children aged six years and older (6).

However, a significant proportion of patients present contraindications, intolerable side effects, or potential risk of misuse associated with stimulant use. In such cases, non-stimulant therapeutic alternatives such as atomoxetine become viable options. Atomoxetine is a selective norepinephrine reuptake inhibitor approved by the U.S. Food and Drug Administration (FDA) for the treatment of ADHD in children and adolescents aged six years and older (7). Unlike stimulants, atomoxetine has no significant abuse potential, making it a preferred choice for individuals with a history of substance use or psychiatric comorbidities (8, 9).

Several international clinical guidelines, including those of the American Academy of Pediatrics (10), recommend atomoxetine as a valid therapeutic option for ADHD treatment, particularly when stimulants are ineffective or contraindicated. Nonetheless, the efficacy and safety of atomoxetine compared with stimulants, particularly in adolescents, remain subjects of debate, as studies have reported varying results regarding the magnitude of clinical response and adverse effect profiles.

Therefore, the objective of this review was to systematically evaluate and summarize the available scientific evidence regarding the efficacy of atomoxetine in reducing ADHD symptoms in adolescents, as well as to investigate adverse events associated with its use compared with other therapeutic options. A critical analysis of the available evidence aims to support data-driven clinical decision-making and optimize ADHD management in this specific population.

## 2 Methods

The systematic literature search followed the *Preferred Reporting Items for Systematic Reviews and Meta-Analyses* (PRISMA) guidelines and the *Cochrane Handbook for Systematic Reviews of Interventions*. This review was registered in the *International Prospective Register of Systematic Reviews* (PROSPERO) under protocol number CRD420251152121, titled “Efficacy and Safety of Atomoxetine in the Treatment of ADHD in Children and Adolescents: A Systematic Review”.

### 2.1 Search strategy

Searches were performed in the following electronic databases: Medical Literature Analysis and Retrieval System Online (Medline, via PubMed), Web of Science, and EMBASE. The search strategies designed and used in each database are presented in Table 1. The initial search was conducted in November 2024 and updated in September 2025.

**Table 1:** Strategies used in the electronic search.

Database	Search strategy	Result**s**
Medline (PubMed)	(atomoxetine hydrochloride) OR (atomoxetine) AND (attention deficit disorder with hyperactivity) OR (ADHD) OR (attention deficit hyperactivity disorder) AND (ADHD symptom reduction) AND (ADHD medications side effects). Filters applied: Randomized Controlled Trial, Child: birth- 18 years	198
EMBASE	(“atomoxetine OR “atomoxetin” OR “atomoxetine hydrochloride”) AND (“attention deficit hyperactivity disorder” OR ‘adhd’ OR ‘attention deficit OR ‘attention deficit and disruptive behavior disorders’ OR ‘attention deficit and disruptive behaviour disorders’ OR ‘attention deficit disorder’ OR ‘attention deficit disorder with hyperactivity’ AND (‘randomized controlled trial’ OR ‘controlled trial, randomized’ OR ‘randomised controlled study’ OR ‘randomized controlled study’.	336
Web of Science	(atomoxetine hydrochloride) OR (atomoxetine) AND (attention deficit disorder with hyperactivity) OR (ADHD) OR (attention deficit disorder) AND (randomized controlled trial) AND (children).	41
Total	–	575

The following descriptors were used: *Children*, *ADHD medications*, *ADHD medications side effects*, *Atomoxetine*, *ADHD symptom reduction*, *Attention-Deficit/Hyperactivity Disorder (ADHD)*, as detailed and presented alongside the search strategy used in Medline via PubMed and adapted for the other databases (Table 1).

### 2.2 Research question

The research question and strategy used in this study were based on the *Population, Intervention, Comparison, Outcome* (PICO) model, commonly applied in evidence-based practice and recommended for systematic reviews.

Accordingly, adolescents with ADHD were considered the *Population*; studies involving the use of atomoxetine as treatment were designated as the *Intervention*; the *Comparison* involved other treatment modalities; and the *Outcome* was the reduction of ADHD symptoms. Thus, the final PICO question was: Is atomoxetine, compared with other treatments, effective in reducing ADHD symptoms in adolescents?

### 2.3 Eligibility criteria

Randomized controlled trials involving children aged 6–16 years were included. Only full-text articles published in English, Spanish, or Portuguese were considered, with no publication year restrictions.

Exclusion criteria included: study designs other than randomized controlled trials; studies involving populations outside the defined age range; and studies that did not report relevant outcomes (efficacy or safety).

### 2.4 Study selection

Study selection was performed independently by one reviewer and conducted in two stages. In the first stage, titles and abstracts of references identified through the search strategy were screened, and potentially eligible studies were preselected. In the second stage, the full texts of preselected studies were reviewed to confirm eligibility. The selection process was conducted using the Rayyan platform (https://rayyan.qcri.org).

### 2.5 Included studies

After the selection process, six randomized controlled trials were included, comprising 905 participants aged 6–16 years. Although the PICO question aimed to compare the efficacy of atomoxetine with other treatments for ADHD in children and adolescents, the included studies predominantly compared atomoxetine with methylphenidate Although our eligibility criteria allowed the inclusion of trials comparing atomoxetine with any active pharmacological treatment, the search identified only randomized controlled trials using methylphenidate as the comparator among adolescents. Therefore, the exclusive presence of methylphenidate-controlled studies reflects the current state of the literature rather than a methodological restriction imposed by the review. And were conducted in China, Taiwan, Turkey, and India (11–16).

### 2.6 Data extraction

Standardized electronic forms were used for data extraction, which included methodological characteristics, interventions, and outcomes. The following study data were collected: authors, publication year, study design, sample size, methods, intervention protocol and control group (if applicable), evaluated outcomes, results, and conclusions.

### 2.7 Assessment of methodological quality

**Figure 1:**
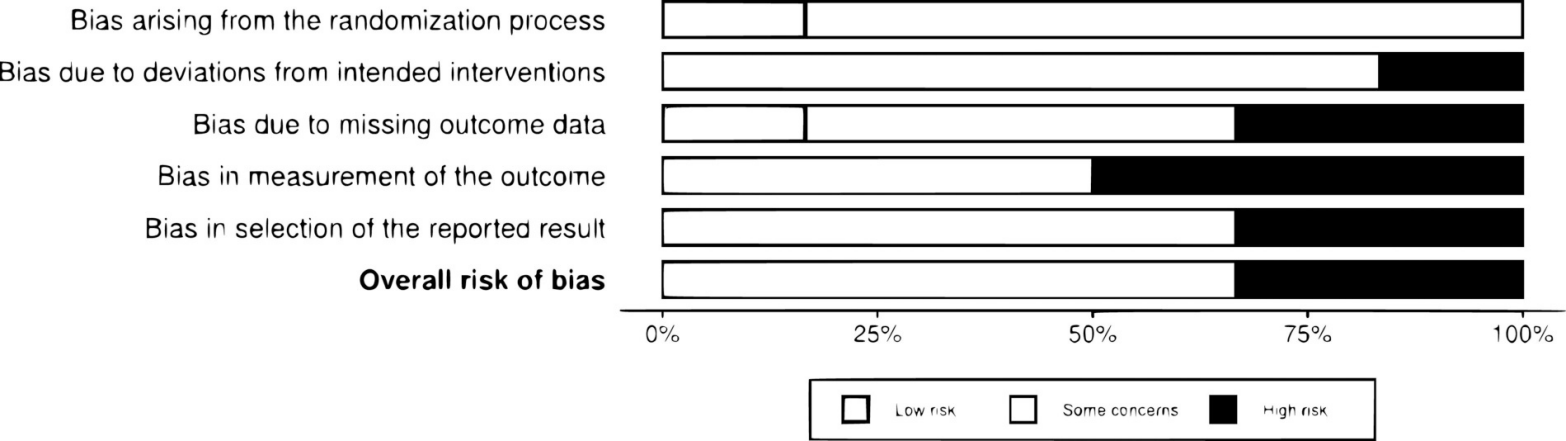
Risk of bias in randomized clinical trials, assessed using the Cochrane risk of bias tool (ROB-2). Adapted from Cochrane (17).

The methodological quality and/or risk of bias of the included studies were independently assessed using tools appropriate for each study design. For randomized controlled trials, the *Cochrane Risk of Bias Assessment Tool* was used. The risk-of-bias assessment for the randomized trials is summarized in Figure 1.

## 3 Results

### 3.1 Search results

**Figure 2:**
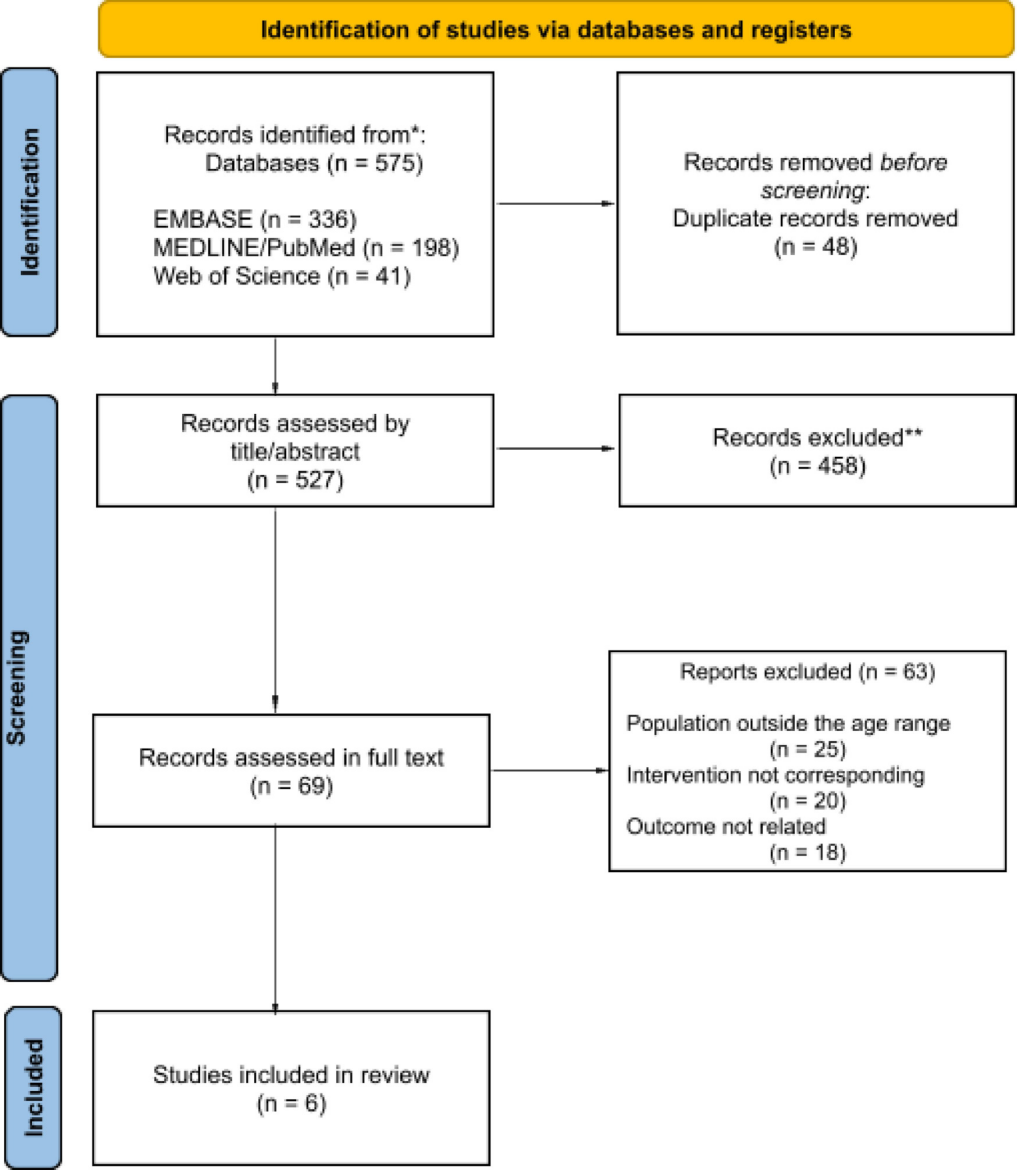
Flowchart of the study selection process (PRISMA flow). Adapted from Page et al. (18).

The database search retrieved a total of 575 records. After removing duplicates, 527 references remained for title and abstract screening, of which 69 met the PICO criteria and were selected for full-text assessment. Following full-text review, 63 articles were excluded due to discrepancies in population, interventions, or reported outcomes. Ultimately, six randomized controlled trials fulfilled the eligibility criteria and were included in the analysis. The study selection process is illustrated in Figure 2, and the characteristics of the included studies are summarized in Table 2.

**Table 2:** Characteristics of included studies.

Study	Design	Participants	Intervention	Duration	Outcome	Results
(11)	RCT	84 children (6–14 years)	ATX vs. MPH	08 weeks	Improvement of symptoms assessed by VADTRS, VADPRS, and CGI-S	Both groups showed a significant reduction (≈25%). Adverse effects: atomoxetine – somnolence, nausea; methylphenidate – insomnia, loss of appetite.
(12)	RCT	160 children (7–16 years)	ATX vs. MPH	24 weeks	Reduction in CGI-ADHD-S and SNAP-IV scores	Average symptom reduction of ≈35% in both groups. No significant differences in overall efficacy. Methylphenidate associated with higher insomnia.
(13)	RCT	262 children (6–16 years)	ATX vs. MPH	08 weeks +1 year	Reduction in ADHD-RS-IV and CGI-ADHD-S scores, tolerability	30% reduction in both groups; lower adherence in the atomoxetine group (60% vs. 75%). Mild adverse effects: atomoxetine – nausea, somnolence; methylphenidate – insomnia, loss of appetite.
(14)	RCT	104 children (6–14 years)	ATX vs. MPH	08 weeks	Reduction in ADHD-RS-IV (Parent) scores, adverse effects	Average reduction of 28% in both groups. Adverse effect profile consistent with the literature; no serious events reported.
(15)	RCT	160 children (7–16 years)	ATX vs. MPH	24 weeks	Improvement of symptoms in CBCL, YSR, and SDQ scores	Overall symptom reduction ≈30%. Methylphenidate superior in reducing aggression and somatic complaints. Adverse effects: atomoxetine – somnolence and nausea; methylphenidate – insomnia and loss of appetite.
(16)	RCT	135 children (6–12 years)	ATX vs. MPH	18 weeks	Improvement in executive functions and reduction in CTRS scores	Average improvement of 30% in executive functions and ADHD symptoms in both groups. Mild adverse effects; no serious events.

RCT, randomized clinical trial; ATX, atomoxetine; MPH, methylphenidate; VADTRS, Vanderbilt ADHD diagnostic teacher rating scale; VADPRS, Vanderbilt ADHD diagnostic parent rating scale; CGI-S, clinical global impression – severity; CGI-ADHD-S, clinical global impression – attention-deficit/hyperactivity disorder – severity; ADHD-RS-IV, attention-deficit/hyperactivity disorder rating scale-IV; CBCL, child behavior checklist; YSR, youth self-report; SDQ, strengths and difficulties questionnaire; CTRS, Conners’ teacher rating scale.

### 3.2 Study results

The six included studies comprised a total sample of approximately 905 participants diagnosed with ADHD, aged between 6 and 16 years. Follow-up durations ranged from eight weeks to twelve months. All trials compared the efficacy and safety of atomoxetine with methylphenidate, in either its immediate-release or osmotic-release formulations. The main assessment scales used to measure outcomes were the ADHD Rating Scale-IV (ADHD-RS-IV), the Clinical Global Impressions-Severity (CGI-S), and the Child Behavior Checklist (CBCL), ensuring standardization and comparability across studies.

In terms of efficacy, results showed that atomoxetine led to an average reduction of 25%–35% in ADHD symptom scores over 8–12 weeks, whereas methylphenidate yielded reductions of 30%–40% within the same period. However, most studies found no statistically significant differences between groups, indicating generally comparable efficacy, with only minor variations in specific domains. Two trials, however, reported modest advantages for methylphenidate: Shih et al. (15) observed greater efficacy in reducing aggression and somatic complaints, while Su et al. (13), in a one-year follow-up, found a sustained response rate of 62% for methylphenidate compared to 54% for atomoxetine. These findings suggest that although overall efficacy is equivalent, methylphenidate may show slightly superior performance in certain behavioral domains and with prolonged use.

Regarding safety and tolerability, adverse effect profiles differed between the medications. Methylphenidate was more commonly associated with insomnia (15%–25%), appetite loss (20%–30%), and weight reduction exceeding 2 kg within up to 12 weeks (10%–15%). In contrast, atomoxetine was more frequently linked to somnolence (10%–20%) and gastrointestinal symptoms such as nausea and vomiting (15%–25%). Treatment discontinuation rates ranged from 12% to 18% for atomoxetine and 8%–15% for methylphenidate, generally due to adverse effects.

Overall, the findings indicate that atomoxetine demonstrates efficacy comparable to methylphenidate in reducing core ADHD symptoms in children and adolescents. However, methylphenidate, considered the first-line treatment by several international guidelines (5, 10), showed a modest advantage in certain behavioral outcomes and higher adherence during longer follow-up periods. Atomoxetine, in turn, stands out as a non-stimulant therapeutic alternative, particularly relevant for patients who cannot tolerate stimulants or have contraindications to their use.

### 3.3 Risk of bias assessment

Overall, according to the Cochrane Risk of Bias 2 (RoB 2.0) tool, none of the studies exhibited a low risk of bias across all domains. Two clinical trials (11, 15) were rated as having a high overall risk of bias, primarily due to lack of blinding, reliance on subjective outcomes reported by parents, substantial loss to follow-up, and failure to apply intention-to-treat analysis in Garg et al. (11).

The remaining studies (12–14, 16) showed “some concerns” in at least one domain, mainly related to the absence of detailed information on allocation concealment, lack of blinding of participants and assessors, and notable sample attrition. Nonetheless, some studies adopted bias-mitigation strategies, such as intention-to-treat analysis (12–14) and the use of objective measures or teacher-reported outcomes (16), which reduced potential expectancy bias from participants or caregivers.

Regarding individual domains, a low risk of bias was generally observed in random sequence generation, although allocation concealment was not clearly described in several studies. Measurement of outcomes and missing data were the domains with the highest number of high-risk judgments due to subjective assessments by unblinded individuals and substantial attrition in some trials, particularly in Shih et al. (15).

In summary, most studies presented a moderate-to-high risk of bias, which must be taken into account when interpreting the results of this systematic review, as illustrated in Figure 1.

## 4 Discussion

The results of this systematic review, which synthesized six randomized clinical trials involving 905 children and adolescents with ADHD, demonstrate that atomoxetine is effective in reducing the core symptoms of the disorder, with overall performance comparable to that of methylphenidate. This consistency, observed across diverse geographic contexts (China, Taiwan, Turkey, and India), reinforces the external validity of the findings.

Although a meta-analysis was initially considered, substantial heterogeneity across the included trials precluded quantitative pooling. The studies used different ADHD symptom rating scales (e.g., ADHD-RS-IV, SNAP-IV, VADTRS), varied dosing strategies for both atomoxetine and methylphenidate, and reported incomplete summary statistics in several cases, particularly missing standard deviations required for effect-size calculation. Follow-up durations also varied considerably, ranging from 8 to 52 weeks, limiting comparability. For these reasons, a narrative synthesis was deemed the most methodologically appropriate approach.

In most studies, no statistically significant differences in efficacy were observed between the two drugs, supporting their clinical equivalence in the short and medium term. Nonetheless, some nuances emerged: methylphenidate showed slightly greater benefits in behavioral outcomes such as reduced aggression and somatic complaints, as well as better adherence over longer follow-up. These findings suggest that while equivalence predominates, subtle differences may guide therapeutic choices in specific cases.

With respect to safety and tolerability, distinct adverse effect profiles were noted. Methylphenidate was more often associated with insomnia, appetite loss, and weight reduction, whereas atomoxetine was linked to somnolence and gastrointestinal symptoms. Nevertheless, none of the studies reported serious adverse events, reinforcing the overall safety of both treatments. Clinical decisions should therefore consider not only efficacy but also individual tolerability profiles and patient characteristics.

An important clinical aspect highlighted in the included trials is that, as a non-stimulant medication, atomoxetine offers specific advantages in ADHD management. Unlike methylphenidate, it has no abuse potential or risk of dependence, making it a safer option for vulnerable populations such as adolescents with a history of substance use. Moreover, several studies included in this review (11–16) emphasized that although its adverse event profile differs from that of methylphenidate, the absence of central stimulation provides benefits for patients with contraindications to stimulants, low tolerance, or heightened risk of insomnia, weight loss, and anxiety symptoms. Thus, atomoxetine represents not only an effective therapeutic alternative but also a strategic option in specific clinical scenarios.

From a clinical standpoint, atomoxetine may be particularly advantageous in certain subgroups. It is considered a preferred option for adolescents with comorbid anxiety disorders, a history of substance misuse or increased risk of stimulant diversion, prominent sleep disturbances, low tolerance to appetite suppression or weight loss, or the presence of motor tics. These scenarios highlight situations in which a non-stimulant profile offers meaningful benefits beyond symptom reduction alone.

Among the limitations of this review are the methodological heterogeneity of the included trials, lack of blinding in some studies, and reliance on caregiver-reported outcomes, which increases the risk of bias. In addition, most studies assessed only short- to medium-term outcomes (8–24 weeks), limiting conclusions about long-term use.

The interpretation of the findings must therefore be viewed in light of these methodological weaknesses. Several trials lacked blinding of participants and outcome assessors, which increases the likelihood of expectancy bias, particularly because many primary outcomes relied on parent-reported symptom scales. In addition, incomplete reporting of allocation concealment in most studies may introduce selection bias, while substantial attrition in two trials raises concerns regarding the robustness and stability of group comparisons. Consequently, although the overall pattern of results indicates comparable efficacy between atomoxetine and methylphenidate, the certainty of this evidence remains moderate at best.

In summary, atomoxetine constitutes an effective and safe alternative for the treatment of ADHD in young individuals. However, further clinical trials with more rigorous methodologies, larger samples, and longer follow-up periods are needed to consolidate its therapeutic role.

## 5 Conclusion

Evidence from the randomized controlled trials included in this systematic review suggests that atomoxetine is an effective and safe option for the treatment of ADHD in children and adolescents, demonstrating overall efficacy comparable to methylphenidate, though with notable differences in tolerability profiles. While methylphenidate was more frequently associated with insomnia and weight loss, atomoxetine presented higher rates of somnolence and gastrointestinal symptoms.

In this context, atomoxetine represents a valid therapeutic alternative, particularly for young individuals with contraindications, poor response, or intolerance to stimulants. Nonetheless, treatment selection should always be individualized, taking into account the patient's clinical profile, comorbidities, and tolerability.

Despite the consistency of findings, this review identified methodological limitations in the evaluated studies, particularly concerning bias risk due to lack of blinding and the predominance of subjective outcomes. Therefore, further randomized clinical trials with larger samples, longer follow-up periods, and stricter methodological rigor are warranted to strengthen the evidence base and better guide clinical practice in managing ADHD in children and adolescents.

## Data Availability

The original contributions presented in the study are included in the article/Supplementary Material, further inquiries can be directed to the corresponding author.
